# Prognostic value of oxygen saturation and heart rate during a six-minute walk test in pediatric pulmonary hypertension

**DOI:** 10.3906/sag-2004-220

**Published:** 2021-08-30

**Authors:** Vildan ATASAYAN, Fatma CANBEYLİ, Fatma Sedef TUNAOGLU, Ayse Deniz OĞUZ, Bülent ÇELİK, Serdar KULA

**Affiliations:** 1 Department of Pediatric Cardiology, Ümraniye Education and Research Hospital, İstanbul Turkey; 2 Department of Pediatric Cardiology, Faculty of Medicine, Kırıkkale University, Kırıkkale Turkey; 3 Department of Pediatric Cardiology, Faculty of Medicine, Gazi University, Ankara Turkey; 4 Department of Statistics, Faculty of Science, Gazi University, Ankara Turkey

**Keywords:** 6-minute walk test, pulmonary arterial hypertension, survival, heart rate

## Abstract

**Background/aim:**

To evaluate the pre and post-treatment heart rate and oxygen (O_2_) saturation values measured before, during and after 6MWT in children who received PAH-specific treatment and to determine the effect of these variables on prognosis.

**Materials and methods:**

This retrospective study included 29 patients who were diagnosed as PAH and treated. The transcutaneous O_2_ saturationand heart rate levels were recorded before the test: baseline (Sat0, HR0), at the end of the test: exercise (Sat1_,_ HR1)and 5 min after the test: recovery (Sat2_, _HR2). The increase in heart rate was defined as HR1-HR0 and the decrease in saturation as Sat0-Sat1. The results obtained before and after the PAH-specific treatment were analyzed and their effect on survival was assessed.

**Results:**

Fifteen of 29 patients were female (51.7%). The mean age was 127.2 ± 44.8 months and the median follow-up time was 60 (12–156) months. Only pre-treatment HR1 was found associated with survival. The mean HR1 value of survivors was 122.8 ± 18.4 and that of deceased 94.3 ± 19.1 (p = 0.03). Post-treatment 6MWT was associated with survival. It was 453.3 ± 96.5 m for survivors and 250 ± 135.2 m for deceased (p = 0.02).

**Conclusion:**

Post-treatment 6MWT, FC and proBNP had prognostic value in pediatric patients with PAH. The decrease in saturation and increase in heart rate were not found to have a prognostic value. Pre-treatment HR1was associated with survival. Identification of these prognostic factors at the beginning and throughout the treatment may be a guide for detecting the severity of the disease and follow-up.

## 1. Introduction 

Until recently, pulmonary hypertension has been widely considered within the context of adult disease. Because of this view, the role of the pediatric disease has been limited and complicated with many unanswered questions throughout the long history of pulmonary hypertension. Finally, the guidelines of the European Society of Cardiology (ESC)/European Respiratory Society (ERS) have underlined the necessity of specific schedules for the diagnosis, follow up and treatment of affected children [1]. After these guidelines of ESC/ERS, two pediatric-specific guidelines were published in a little while [2,3]. However, there are still different opinions about the value of some prognostic factors in both pediatric guidelines [4]. The 6-minute walk test (6MWT) is one of them.

6MWT is effective in predicting survival and useful as a prognostic factor in adults [5,6]. However, the prognostic value of 6MWT is controversial in children due to the difficulty of its feasibility and lack of sufficient data and studies have varying results [7–12]. Although the degree of desaturation and heart rate variations during 6MWT have been shown to be an additional predictor factor in adults with pulmonary arterial hypertension (PAH) [13–15], few studies are investigating this in children [16]. 

In this study, our objective was to evaluate the pre and post-treatment heart rate and oxygen (O_2_) saturation values measured before, during and after 6MWT in children who received PAH-specific treatment and to determine the effect of these variables on prognosis.

## 2. Materials and methods

This retrospective study included 29 patients who were eligible to undergo a 6MWT out of 41 patients diagnosed as PAH and treated at the Pediatric Cardiology Clinic of University Hospital. 

Inclusion criteria were; ≥ 7 and < 18 years of age, diagnosis of PAH either secondary to congenital heart disease or idiopathic, and eligibility to undergo 6MWT. 

Exclusion criteria were; children with a chromosomal abnormality and genetic disorders, inability to perform 6MWT due to physical or mental retardation. 

The criteria for PAH diagnosis were; mean pulmonary arterial pressure (mPAP) ≥ 25 mmHg during a heart catheterization, pulmonary capillary wedge pressure (PCWP) ≤ 15 mmHg, and pulmonary vascular resistance index (PVRI) ≥ 3 Wu.m^2^ [17]. The test was conducted according to the guidelines reported by the American Thoracic Society (ATS) [18]. The patients’ demographic characteristics, etiologies of PAH, WHO functional classes (FC), and PAH-specific treatment modalities were recorded. ProBNP (brain natriuretic peptide) levels were measured. The patients were assessed by their PAH follow-up protocols during their quarterly follow-up and 6MWT was performed in each follow-up visit. The transcutaneous O_2_ saturation and heart rate levels were recorded before the test: baseline (Sat0, HR0), at the end of the test: exercise (Sat1, HR1) and 5 min after the test: recovery (Sat2, HR2). Heart rate and transcutaneous O_2_ saturation were measured with a hand-held pulse oximeter (PC-60B, China) placed on the patient’s right-hand index finger. 

The increase in heart rate was defined as HR1-HR0 and the decrease in saturation as Sat0-Sat1. The results obtained before and after the PAH-specific treatment were analyzed and their effect on survival was assessed. 

### 2.1. Statistical analysis

All statistical analyses were performed using the SPSS (Statistical Package for Social Sciences) v: 15.0 (SPSS Inc, Chicago, IL). Continuous data were presented as means with standard deviation or medians with minimum and maximum values. Categorical data were presented as frequencies and percentages. Continuous variables were tested for normal distribution using the Kolmogorov–Smirnov test. Two dependent groups were compared using the paired sample t-test for normal distributions and the Wilcoxon test for nonnormal distributions. Correlation coefficients (r) were calculated using the Pearson correlation test or Spearman’s rank test, depending on the normality of the p-value. Kaplan–Meier curves with log-rank tests were plotted for the patients who scored below and above the median 6MWT. Univariate survival analysis was performed using the Cox-regression test. A two-sided p-value < 0.05 was considered statistically significant for all analyses. An a priori power analysis was conducted in G*Power Software (v: 3.1.9.5; Franz Faul, Universität Kiel, Germany). The sample size calculation was based on the mean 6MWT values derived from previous research and an alpha significance level set at 0.05, to achieve 80% statistical power approximately 28 individuals would have to be recruited [19].

## 3. Results

Fifteen of 29 patients were female (51.7%). The mean age was 127.2 ± 44.8 months and the median follow-up time was 60 (12–156) months. Two patients (6.9%) were diagnosed with idiopathic PAH and 27 patients (93.1%) with secondary PAH. The ventricular septal defect was the most common (38%) congenital heart disease among the etiologies of secondary PAH. All patients underwent cardiac catheterization. The comparison of patients’ clinical characteristics between before and after treatment were shown in Table 1. 

**Table 1:** Comparison of variables before and after treatment.

Variables		Before treatment	After treatment	P-value
proBNP (pg/mL)*		218 (20–9000)	186 (10–7958)	0.112
6MWT (m)*		390 (120–624)	441 (120–603)	0.026
Mean PAP (mmHg)*		53 (15–110)	71.5 (34–137)	0.003
Saturation (%) +	0	87.3 ± 12.3	86.6 ± 9.1	0.470
	1	76.2 ± 21.6	65.0 ± 26.7	0.003
	2	81.8 ± 16.6	71.7 ± 21.3	0.017
HR (beat/min)+	0	96.5 ± 15.9	95.2 ± 13.7	0.810
	1	118.9 ± 20.4	132.3 ± 22.7	0.017
	2	106.1 ± 17.4	114.5 ± 21.4	0.260
FC (%)	I		13.8	
	II	20.7	55.2	
	III	62.1	24.1	
	IV	17.2	6.9	
Monotherapy (n)		11	9	
Combined therapy (n)		18	17	

*Data were presented as median (min-max), +Data were presented as mean ± standard deviation (SD). proBNP: brain natriuretic peptide, m: meter, 6MWT: 6-minute walk test, PAP: pulmonary arterial pressure, HR: heart rate, FC: functional class.

### 3.1. Heart rate and saturation assessment

The post-treatment HR1 was significantly higher than pre-treatment (132.3 ± 22.7, 118.9 ± 20.4, p = 0.017), but there was no significant difference in HR0 and HR2 (Table 1). The pre-treatment Sat1 and Sat2 were significantly higher than post-treatment (p = 0.003, p = 0.017) (Table 1). 

### 3.2. Comprehensive 6MWT evaluation and correlations

The pre- and post-treatment 6MWT had a significant negative correlation with both FC and proBNP (p = 0.004, p < 0.001; p = 0.046, and p = 0.003 respectively) (Table 2).

**Table 2:** Correlations between variables before and after treatment.

Variables			Before treatment			After treatment		
			6MWT (m)	proBNP(pg/mL)	FC	6MWT (m)	proBNP (pg/mL)	FC
proBNP (pg/mL)		Corr. coef.	–0.373	1	0.287	–0.526	1	0.527
		P-value	0.046		0.131	0.003		0.003
FC		Corr. coef.	–0.520	0.287	1	–0.672	0.527	1
		P-value	0.004	0.131		<0.001	0.003	
Saturation (%)	0	Corr. coef.	0.448	–0.150	–0.338	0.400	–0.101	–0.507
		P-value	0.015	0.437	0.073	0.035	0.609	0.006
	1	Corr. coef.	0.459	–0.016	–0.312	0.320	–0.084	–0.443
		P-value	0.012	0.936	0.100	0.096	0.669	0.018
	2	Corr. coef.	0.654	–0.195	–0.490	0.535	–0.170	–0.546
		P-value	<0.001	0.351	0.013	0.004	0.395	0.003
HR (beat/min)	0	Corr. coef.	–0.374	–0.023	0.095	–0.436	0.191	0.056
		P-value	0.046	0.906	0.625	0.020	0.330	0.777
	1	Corr. coef.	–0.131	0.060	0.012	0.089	–0.105	–0.113
		P-value	0.497	0.759	0.951	0.654	0.596	0.567
	2	Corr. coef.	–0.438	0.054	0.118	–0.190	0.018	0.092
		P-value	0.029	0.798	0.576	0.342	0.929	0.648

Corr. coef: correlation coefficient, FC: functional class, proBNP: brain natriuretic peptide, SAT0: baseline O2 saturation before the test, SAT1: O2 saturation at the end of the test, SAT2: O2 saturation 5 min after the test, HR0: baseline heart rate before the test, HR1: heart rate at the end of the test, HR2: heart rate 5 min after the test.

Pre-treatment 6MWT had a significant positive correlation with Sat0, 1, 2 (p = 0.015, p = 0.012, p < 0.001) while post-treatment 6MWT had with only Sat0 and Sat2 (p = 0.035, p = 0.004).

Pre-treatment 6MWT was negatively correlated with HR0 and HR2 (p = 0.046, p = 0.029) and post-treatment 6MWT with only HR0 (p = 0.037). 

Pre-treatment FC had a significant negative correlation with only Sat2 and post-treatment FC with all Sat0, 1, and 2. While desaturation had no correlation with proBNP, it had a significant positive correlation with post-treatment FC (p = 0.04, r = 0.0378).

There was no correlation between Sat0, 1, 2, and proBNP before or after the treatment. 

Heart rate increases, HR0, 1, 2 were not correlated with pre- and post-treatment FC or proBNP. 

There was no significant correlation between pre-treatment FC and proBNP, but a statistically significant positive correlation was found after the treatment (p = 0.003). 

### 3.3. FC III-IV versus FC I-II

While 16.7% of the 6 patients who were FC I -II at the initial assessment regressed to FC III-IV after the treatment, 65.2% of the 23 patients who were FC III-IV at baseline improved to FC I-II and 34.8% of them remained as FC III-IV (p < 0.001). In the final assessment, the 6MWT distance and proBNP of the patients who were FC III-IV were significantly higher than those whom FC I-II (p = 0.003; p = 0.020, respectively) (Table 3). Only the post-treatment Sat2 value was significantly lower in those who were FC III-IV (p = 0.033) (Table 3). Pre- and post-treatment HR0, 1, and 2 did not differ between FC III-IV and FC I-II. Pre-treatment heart rate increase was found significantly higher in those who were FC I-II (p = 0.027) (Table 3). 

**Table 3:** Comparison of variables between FC III-IV and FC I-II before and after treatment.

Variables		Before treatment																
		FC I - II (n: 6)				FC III - IV (n: 23)				P-value	FC I - II (n: 20)			FC III - IV (n: 9)				P-value
proBNP(pg/mL)		87.5	(20.0–		1423.0)	282.0	(51.3	-	9000.0)	0.027	98.3	(23.9	823.0)	1337.0	(10.0	-	7958.0)	0.020
6MWT(m)		453.5	(304.0	-	–624.0)	377.0	(120.0	-	564.0)	0.085	486.0	(300.0	603.0)	345.0	(120.0	-	500.0)	0.003
Saturation(%)	0	97.0	(61.0	-	99.0)	90.0	(59.0	-	99.0)	0.099	93.0	(70.0	99.0)	83.0	(69.0	-	97.0)	0.052
	1	93.5	(48.0	-	97.0)	83.0	(25.0	-	98.0)	0.215	83.0	(16.0	98.0)	48.0	(17.0	-	97.0)	0.061
	2	96.0	(52.0	-	99.0)	86.0	(54.0	-	98.0)	0.056	94.0	(34.0	99.0)	53.5	(52.0	-	78.0)	0.033
	Decrease	3.0	(1.0	-	18.0)	7.0	(-3.0	-	48.0)	0.318	12.0	(-4.0	64.0)	27.0	(0.0	-	62.0)	0.140
HR(beat/min)	0	98.0	(65.0	-	128.0)	95.0	(79.0	-	123.0)	0.957	97.0	(57.0	120.0)	95.0	(80.0	-	114.0)	0.999
	1	124.0	(100.0	-	160.0)	116.0	(73.0	-	153.0)	0.319	141.0	(103.0	185.0)	128.0	(100.0	-	160.0)	0.459
	2	108.0	(83.0	-	134.0)	104.0	(80.0	-	137.0)	0.799	113.0	(71.0	164.0)	109.0	(80.0	-	142.0)	0.894
	Increase	43.0	(-13.0	-	47.0)	19.0	(-19.0	-	48.0)	0.027	40.0	(10.0	83.0)	23.0	(10.0	-	67.0)	0.313

Data were presented as median (min-max), FC: functional class, proBNP: brain natriuretic peptide, SAT0: baseline O2 saturation before the test, SAT1: O2 saturation at the end of the test, SAT2: O2 saturation 5 min after the test, HR0: baseline heart rate before the test, HR1: heart rate at the end of the test, HR2: heart rate 5 min after the test.

### 3.4. Survival factors

During follow-up, 3 patients in FC III-IV have died. The first patient who was followed up for patent ductus arteriosus-related PAH died due to a serious lung infection. The second and third patient diagnosed as residual and idiopathic PAH were lost from heart failure. Only pre-treatment HR1 was found associated with survival. The mean HR1 value of survivors was 122.8 ± 18.4 and that of deceased 94.3 ± 19.1 (p = 0.03) (Table 4). 

**Table 4:** Parameters associated with patient survival.

	N	Deathn (%)	Univariate analysis		
			HR	95% CI	P-value
At diagnosis WHO-FC					
I-II	6	-	1		
III-IV	23	3 (13.0)	0.513	0.053–4.955	0.564
proBNP			1.001	1.000–1.002	0.066
6 MWT			1.071	0.918–1.250	0.381
Sat0			0.990	0.979–1.001	0.064
Sat1			1.096	0.948–1.268	0.217
Sat2			1.035	0.896–1.196	0.638
Sat decrease			0.759	0.560–1.029	0.076
HR0			0.960	0.885–1.040	0.317
HR1			0.939	0.885–0.996	0.036
HR2			0.730	0.406–1.312	0.293
HR increase			0.949	0.894–1.006	0.080
At last evalution					
WHO-FC					
I-II	20	-	1		
III-IV	9	3 (33.3)	9.393	0.975–55.492	0.049
proBNP			1.001	1.000–1.001	0.004
6 MWT			0.983	0.969–0.998	0.025
Sat0			1.039	0.902–1.196	0.596
Sat1			1.027	0.975–1.083	0.315
Sat2			0.991	0.916–1.072	0.822
Sat decrease			0.955	0.876–1.042	0.301
HR0			1.033	0.934–1.143	0.526
HR1			0.991	0.941–1.043	0.724
HR2			0.983	0.914–1.058	0.650
HR increase			0.976	0.912–1.044	0.478

WHO-FC: World Health Organization-Functional Class, proBNP: brain natriuretic peptide, SAT0: baseline O2 saturation before the test, SAT1: O2 saturation at the end of the test, SAT2: O2 saturation 5 min after the test, HR0: baseline heart rate before the test, HR1: heart rate at the end of the test, HR2: heart rate 5 min after the test.

**Figure 1:**
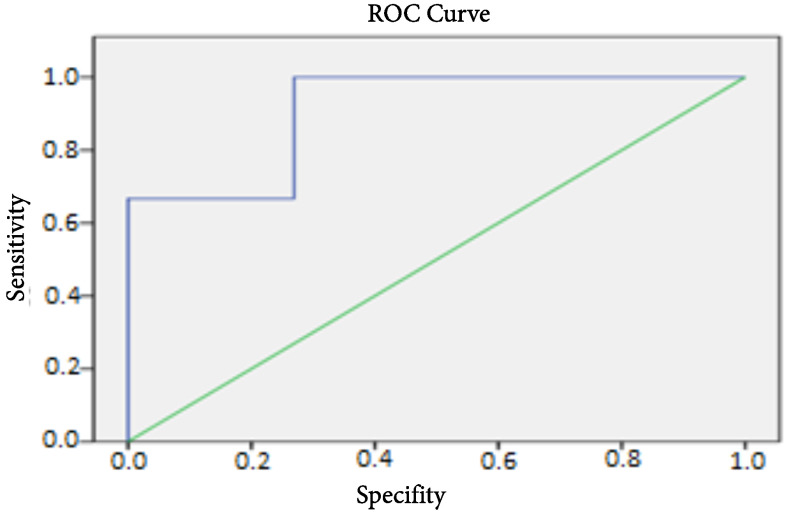


**Figure 2:**
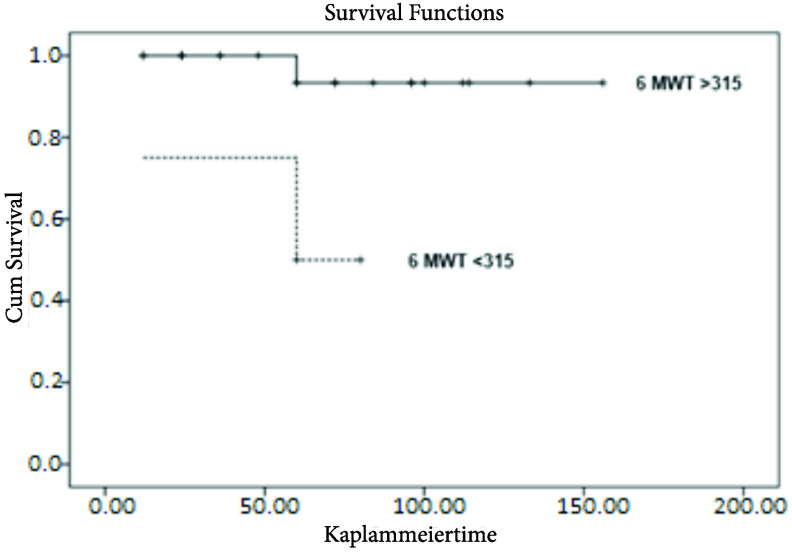


Post-treatment 6MWT was associated with survival. It was 453.3 ± 96.5 m for survivors and 250 ± 135.2 m for the deceased (p = 0.02) (Table 4). A ROC analysis revealed that the cut-off value for 6MWT was 315 m with 66.7% sensitivity and 92.3% specificity (Figure 1). The survival time was 53 ± 28.9 months for whom 6MWT < 315 m and 64.9 ± 41.1 months for whom 6MWT > 315 m (p = 0.01) (Figure 2). A high level of post-treatment proBNP affected survival negatively (p = 0.004). Similarly, post-treatment FC being III-IV had a 9-fold negative effect on survival (Table 4). 

## 4. Discussion

Our study showed that pre- and post-treatment 6MWT in pediatric patients with pulmonary arterial hypertension was negatively correlated with FC and proBNP and all of these three parameters were associated with post-treatment survival. A high pre-treatment heart rate (HR1) as measured at the end of the test was found to have a positive impact on survival. The saturation decrease and heart rate increase during the test seemed to have no prognostic effect on survival. 

Studies investigating the prognostic value of 6MWT in pediatric patients with PAH have conflicting results [7–12]. Ivy et al. have stated that unlike in adults, 6MWT should not be used in children with PAH for risk assessment [7]. Studies are having similar results in the literature [20,21]. Whereas, Lammers et al. reviewed the data of 47 children with PAH with a mean age of 11.4 years and reported that 6MWT as a predictor factor for death or need for transplantation [10]. Our study also revealed that there was a statistically significant increase in post-treatment 6MWT distance and post-treatment test was associated with survival. Additionally, the fact that 6MWT had a significant negative correlation with FC and proBNP as two important predictor factors before and after the treatment, showed that this test could be used to evaluate the prognosis and severity of the disease. The cut-off point for the test was found to be 315 m in our study and those whose 6MWT distance > 315 m were shown to have better survival. The test can also be used to evaluate the quality of life and the effort capacity of patients irrespective of survival. 

Recovery of heart rate at the first minute has been shown to be a predictor factor in adults with PAH. Ramos et al. have reported that adult patients whose recovery of heart rate at the first minute > 18 pulses have better hemodynamic outcomes, NYHA scores and 6MWT distances. Because the autonomic nervous system dysfunction in patients with PAH increases morbidity and mortality [13,15]. In Douwes et al.’s study with 47 pediatric patients with PAH (15 with PAH-CHD and 32 with IPAH), recovery of heart rate at the fifth minute and increase in heart rate had no prognostic value in pediatric patients with PAH [16]. In our study, pre-treatment HR1 was associated with survival, and similar to Douwes et al., pre- or post-treatment heart rate increase was shown not to affect survival [16]. Although our study showed that post-treatment HR1 did not affect survival, having a higher HR1 after the treatment may indicate a better response to exercise due to the partial improvement of post-treatment cardiac autonomic functions.

As in the study of Douwes et al., our study also demonstrated that the post-treatment saturation at the end of the exercise (Sat1) had a negative correlation with WHO-FC, the post-treatment desaturation rate had a positive correlation with FC and neither of these two had any correlation with proBNP [16]. Even though Sat1 and desaturation rate did not affect survival, their correlation with post-treatment FC, which is a factor for survival, may provide hints on prognosis.

Zuk et al. reported 6MWT was negatively correlated with the maximum heart rate, but there was no correlation between these two parameters in our study [19]. The presence of a shunt in pediatric patients with PAH was associated with shorter 6MWT distance, lower saturation at rest and higher desaturation rate after effort [19]. Lammers et al. [22] and Douwes et al. [16] have also reported similar results. Impaired exercising capacity and decreased saturation in PAH patients without a shunt are thought to be associated with decreased cardiac output due to right ventricle impairment and ventilation-perfusion mismatch due to gas diffusion disorder at the pulmonary vascular bed [23]. In patients with PAH having a shunt, the cardiac index during exercise increases or is maintained at the same level depending on the increase in the right-to-left shunt and this results in a further decline in saturation in these patients. Since there were only 2 patients with IPAH in our study, the comparison of these 2 groups couldn’t be done. 

Although Douwes et al. found that the decrease in saturation during 6MWT was associated with poor survival in patients having a shunt, our study showed no association [16]. Zuk et al. also reported the presence of higher desaturation during effort in patients having a shunt but did not evaluate its association with survival [19]. The results may have differed because, unlike other studies, a large majority of the patients in our study consisted of patients with PAH secondary to congenital heart disease. In patients with Eisenmenger syndrome, decreased right ventricle workload due to increased right-to-left shunt during exercise, presence of an escape for compensation and maintaining the cardiac index as a result of these have beneficial effects, but our study did not show any effect of the desaturation rate on their survival in these patients. 

Our study showed that patients who were FC III-IV had shorter 6MWT distance and higher proBNP levels both before and after the treatment compared to those who were FC I-II. These results are similar to Zuk et al. study [19]. However, Zuk et al. reported resting and peak HR were significantly higher in patients who were FC III-IV, but they found no difference in resting saturation and desaturation [19]. Our study did not show any difference between these two groups in terms of heart rate. The pre-treatment heart rate increase was significantly higher in patients who were FC I-II and the post-treatment Sat2 significantly lower in those who were FC III-IV. 

We didn’t find any prognostic effect of mean PAP on survival similarly to the literature. [24]. Also, there was an increase in mean PAP after PAH-specific treatment in our study. It is well known that PAH is a progressive disease. Therefore, an increase in mean PAP is an expected result in patients with PAH, especially in IPAH. However, the effect of PAH-specific treatments on mean PAP remains unclear [25–27]. 

The limitations of our study include; a small number of patients and the inability to make a comparison between IPAH and secondary PAH because the majority of our participants consisted of patients with PAH secondary to congenital heart disease. Additionally, the heart rate at the first minute which has been shown to have a prognostic value in adult patients with PAH was not evaluated [13,15]. 

In conclusion, this study showed that post-treatment 6MWT, FC and proBNP had prognostic value in pediatric patients with PAH. The decrease in saturation and increase in heart rate were not found to have a prognostic value. Pre-treatment HR1 was associated with survival. Identification of these prognostic factors at the beginning and throughout the treatment may be a guide for detecting the severity of the disease and follow-up.

## Ethical approval 

Ethical approval was obtained from the Clinical Trials Ethics Committee of the Gazi University.

## Informed consent

Informed consent was obtained from all individual participants or their parents included in the study.
